# 885. Burn Center Mortality, Can We Do Better?

**DOI:** 10.1093/jbcr/irag033.371

**Published:** 2026-04-06

**Authors:** Susan Hecker, Heidi M Altamirano, Mark J Johnston, Alexandra M Lacey

**Affiliations:** Regions Hospital, Saint Paul, MN; Regions Hospital Burn Center, Saint Paul, MN; Regions Hospital, Saint Paul, MN; Regions Hospital, Saint Paul, MN

## Abstract

**Introduction:**

Burn care has advanced in ways that early practitioners could not predict. The team approach in centers of excellence contributes to the survival of patients with the most grievous injuries. Best practices are validated in the literature, and treatment plans are implemented based upon reduction of mortality. In our burn center, we have adopted protocols derived from best practices, with survival reaching 98% in 532 patients treated in the past year. We chose to review the patients who succumbed to their injuries to identify opportunities to improve the care of these patients as well as the support provided to regional referral sites.

**Methods:**

A retrospective chart review was conducted from July 1, 2024-June 30, 2025. All burn patients that died during this period were analyzed. Variables included patient and burn characteristics, hospital transfer, timeliness of goals of care conversations, palliative care consultation, and others.

**Results:**

Eleven deaths occurred during this period, with age ranging from 39-91 years. Nine were flame burns, two of which were self-immolation, one had hypothermia and frostbite, while another had a small contact burn with acute medical issues. The Revised Baux Score ranged from 109-146. Two patients arrived from local EMS in PEA arrests and died. Six patients had inhalation injuries while two had associated traumatic injuries. Six patients were referred from rural facilities, four of which transitioned to comfort care within 24 hours of arrival. Goals of care conversations consistently occurred early on, with the provider, social worker, therapist, and chaplain, while palliative care consultation occurred in three cases.

**Conclusions:**

This comprehensive review of burn mortality reinforced the need for multidisciplinary support in this population. We were unable to determine appropriateness of transfer due to a limited number of patients as well as factors including lack of knowledge of comorbidities, complex social situations, and unknown prognosis of acute medical issues. With regionalization of expertise to centers of excellence and uncertainty of local patient evaluation it is reasonable to attempt transfer. Creating a guideline outlining timing of palliative care consultation allows for decisional flexibility when caring for burn patients. Future work includes partnering with referral sites through a regional telemedicine program to ensure the discussion of burn survivability includes associated factors such as acute medical or traumatic injuries and comorbidities.

**Applicability of Research to Practice:**

Patients transferred from regional facilities who transition to comfort care early in the hospital course is scrutinized. This project identified factors to consider when accepting transfers from rural facilities while also realizing the desire for burn expertise to assist in this difficult decision. Further work will be done to identify opportunities to support both the patient and family, and regional care system.

**Funding for the study:**

N/A.

